# Effects of the RNA-Polymerase Inhibitors Remdesivir and Favipiravir on the Structure of Lipid Bilayers—An MD Study

**DOI:** 10.3390/membranes12100941

**Published:** 2022-09-27

**Authors:** Mauro Bringas, Meike Luck, Peter Müller, Holger A. Scheidt, Santiago Di Lella

**Affiliations:** 1 Instituto de Química Biológica—Ciencias Exactas y Naturales (IQUIBICEN)—CONICET and Departamento de Química Biológica FCEN, Universidad de Buenos Aires, Int. Güiraldes 2160, Buenos Aires C1428EGA, Argentina; 2 Department of Biology, Humboldt Universität zu Berlin, Invalidenstr. 42, D-10115 Berlin, Germany; 3 Institute for Medical Physics and Biophysics, Leipzig University, Härtelstr. 16–18, D-04107 Leipzig, Germany

**Keywords:** remdesivir, favipiravir, membrane, molecular dynamics simulation, all-atom, antivirals

## Abstract

The structure and dynamics of membranes are crucial to ensure the proper functioning of cells. There are some compounds used in therapeutics that show nonspecific interactions with membranes in addition to their specific molecular target. Among them, two compounds recently used in therapeutics against COVID-19, remdesivir and favipiravir, were subjected to molecular dynamics simulation assays. In these, we demonstrated that the compounds can spontaneously bind to model lipid membranes in the presence or absence of cholesterol. These findings correlate with the corresponding experimental results recently reported by our group. In conclusion, insertion of the compounds into the membrane is observed, with a mean position close to the phospholipid head groups.

## 1. Introduction

Cellular membranes are essential for life. They are responsible for preserving the homeostatic environment within the cell and maintaining crucial cellular functions [1]. As an interface that separates the extracellular medium from the intracellular one, membranes are decisive for processes such as the uptake of extracellular molecules by endocytosis or the permeation of ions across membrane channels. Likewise, drugs, which are applied for the therapeutic treatment of diseases, come into contact with the plasma membrane and have to enter cells by crossing the membrane to exert their functions. Research was mainly focused on the relationship between drugs and their cognate protein receptors, without delving much into the interaction with membranes. However, mechanisms of action of many drugs cannot be solely explained by their specific effects; therefore, a considerable interest in investigations of the drug-related impacts affecting the membrane has evolved in recent years [2,3,4,5]. The knowledge of these mechanisms may help improve the efficacy of drugs, e.g., by enhancing their cellular uptake or understanding and reducing unwanted side effects.

Membranes are assemblies of molecules forming supramolecular structures whose properties depend on a multitude of factors such as composition, temperature, lateral and hydrostatic pressure, ion concentration, pH, and the presence of ligands such as drugs or proteins [6]. Even small environmental changes can cause altered membrane properties. However, an exact assignment of these changes to distinct molecular processes is often difficult to study experimentally, especially on an atomic scale. Nowadays, a set of computational tools is available, such as molecular dynamics simulation (MD), which can help in understanding the dynamics of different complex membrane systems [7,8,9]. In particular, we are able to study the influence of drug molecules on the structure and dynamics of membranes at the atomistic level. These properties include, for example, changes in the melting point that would be caused by different drug concentrations in the liquid/gel phases of the membrane, which translate into differences in the osmotic pressure and in the lateral pressure profile of the membrane [10]. The results of MD studies deepen our understanding of the molecular mechanisms underlying the mechanisms of action of molecules displaying different chemical structures commonly used as drugs and help in proposing directions for further drug development [2,11,12,13].

**Figure 1:**
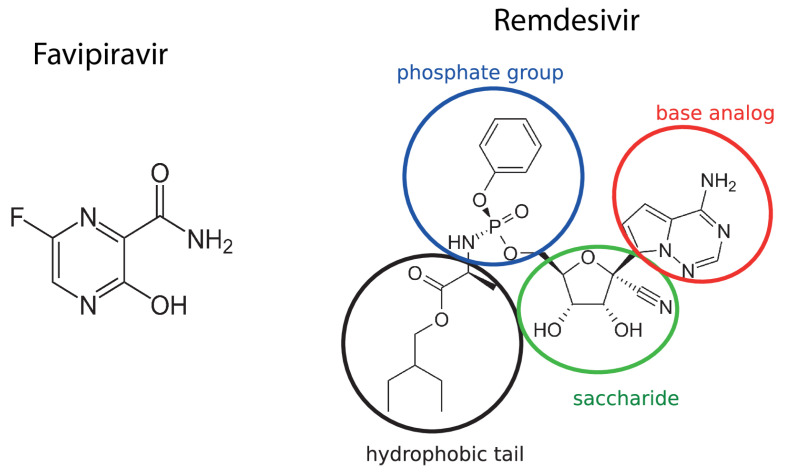
Chemical structure of favipiravir and remdesivir.

With the emergence of the current COVID-19 pandemic, the urgent need for applicable antivirals was impressively enhanced. Since the development and approval of new drugs are time-consuming processes, a variety of already existing drugs have been tested for their potential use against coronavirus. For the latter, specific nucleoside analogues were developed as inhibitors of the virus-specific RNA-dependent RNA polymerase (RdRp), an enzyme which is also involved in the replication of the SARS-CoV-2 genome. Two existing RdRp inhibitors (broad-spectrum antivirals), remdesivir and favipiravir, were repurposed as a fast therapeutic approach for an immediate treatment of COVID-19 [14,15]. Remdesivir (structure, see Figure 1) is a monophosphoramidate nucleoside analogue that was primarily developed for the treatment of filoviral ebola infections and has been proven to inhibit in vitro SARS-CoV-1, MERS-CoV, and SARS-CoV-2. Favipiravir (structure, see Figure 1) is a guanine analogue that was previously applied as a drug against influenza and ebola [16]. Both molecules are prodrugs that are metabolized upon administration into the physiologically active nucleotide analogues inhibiting the SARS-CoV2 RdRp [17,18]. While studies generated promising results for both drugs in the treatment of SARS-CoV-2 infections in in vitro experiments, their grade of efficacy in therapeutics is still unclear and contradictory partially because of an insufficient number of studies and partially due to unclear outcomes and side effects of the treatments. In order to understand the efficacy and also the side effects of the drugs on a molecular level, it is important to determine their influence on the structure and dynamics of cell membranes. Therefore, we have recently investigated the impact of remdesivir and favipiravir on the lipid bilayer in model and cell membranes using several biophysical approaches [19]. The measurements revealed that remdesivir incorporates into the bilayer causing a disturbance of the membrane structure. In contrast, for favipiravir, while also inserting into membranes, no indications for a membrane impact were found. Extending this experimental study, in the present work we applied MD simulations to further elucidate the interaction of both drugs with membranes.

## 2. Computational Methods

We prepared 1-palmitoyl-2-oleoyl-sn-glycero-3-phosphocholine (POPC) model membranes using CHARMM-GUI [20]. A POPC membrane system was created containing 128 lipid molecules which were solvated with 5000 water molecules and Na^+^/Cl^−^ ions (final concentration 0.15 M) to represent the experimental conditions. Additionally we prepared a POPC:Cholesterol (0.8:0.2 molar ratio) mixture membrane containing a total of 128 lipid molecules with the same amount of solvent and ionic species. Initial configurations for each simulated system as well as final membrane composition details are shown in Supplementary Materials, Figures S1 and S2 and Table S1. For MD simulations, we employed the AMBER 18 package of programs [21]. The tleap module was used to add parameters for lipid molecules (Lipid21 forcefield) [22], water molecules (TIP3P model) [23], and ions [24]. The system was subjected to 5000 steps of steepest descent minimization followed by 5000 steps of a conjugated gradient. Subsequently, the system was linearly heated up to 303 K in the NVT ensemble using a Langevin thermostat for 1 ns, maintaining harmonic restrictions on all lipid molecules with a spring constant of 10.0 kcal/Å^2^. The system was then allowed to relax in the NPT ensemble in 10 short phases without restrictions for a total of 5 ns using a Berendsen barostat with anisotropic scaling followed by 500 ns production dynamics at the same conditions.

**Scheme 1:**
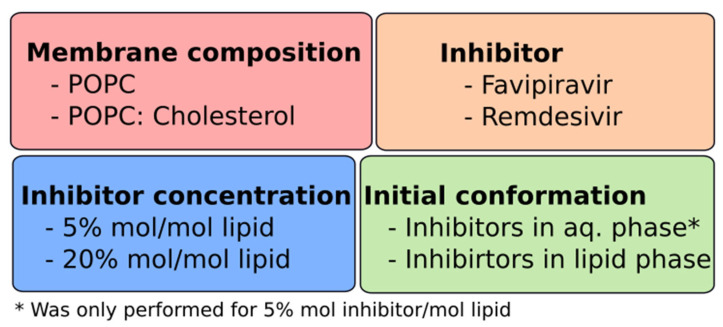
Conditions explored for the molecular systems tested. Each possible combination for the conditions was tested, yielding 12 systems for MD simulation production. For details, see Figures S1 and S2 and Table S1 in the Supplementary Materials.

Remdesivir and favipiravir parameters were obtained using a standard protocol. We calculated RESP charges using a Hartree–Fock single point calculation with 6–31G* basis set. Bond, angle, and dihedral constants, together with atom types, were assigned from other moieties in the GAFF Forcefield using Antechamber and Parmcheck modules. For modeling membrane systems in the presence of inhibitors, a membrane was first set up using the same procedure as the one for the pure membrane described above. Subsequently, the system was desolvated and a defined number of molecules of either remdesivir or favipiravir were introduced using a Packmol [25]. In order to test if the final configuration of the molecules was affected by the initial distribution, we incorporated drug molecules in two different ensembles: at random positions in the aqueous phase or at random positions in between the leaflets of the lipid bilayer. The initial conformations of the inhibitor molecules were selected randomly from a previous implicit solvent trajectory. A total of 5000 and 8000 TIP3P water molecules were added to re-solvate the system for favipiravir and remdesivir, respectively, preventing solvent incorporation in the lipid phase. Similar minimization, thermalization, and relaxation protocols were employed as described above. To consider two different drug concentration conditions, we generated simulation boxes containing 5% and 20% mol inhibitor/mol lipid. As stated before, drug molecules were inserted either in the aqueous phase or inside the bilayer under low concentration conditions and in the lipid phase under high concentration conditions. 3 replicates of 400 ns each and production MD were performed for each of the 12 different ensembles presented in Scheme 1.

## 3. Results

### 3.1. Drugs Insertion into the Membrane

**Figure 2:**
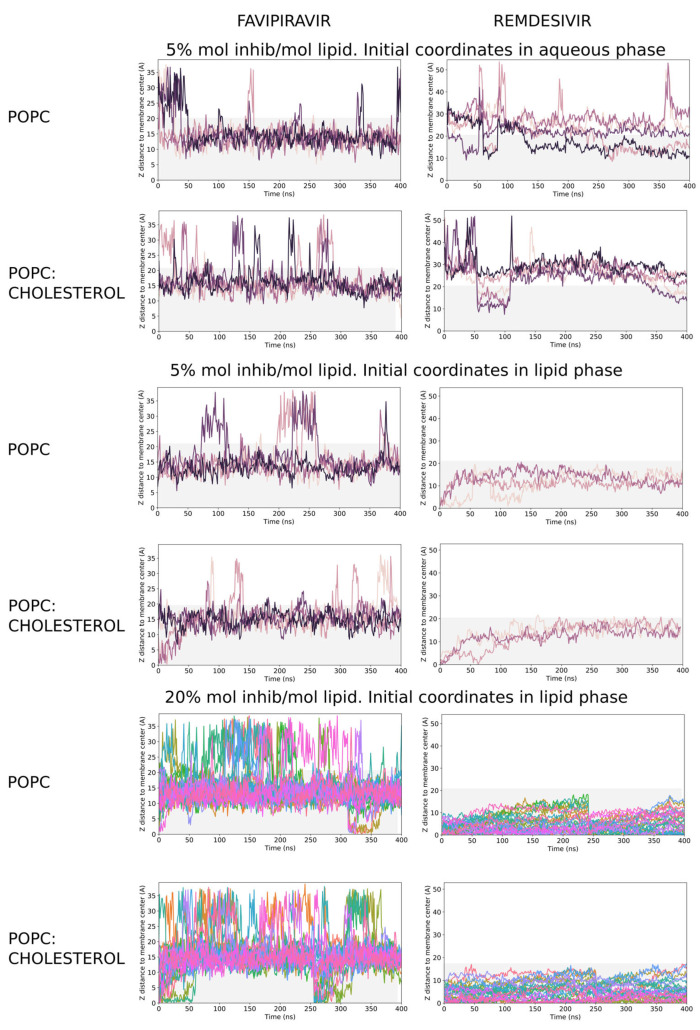
Position of the center of mass as a function of the simulation time of favipiravir (**left**) and remdesivir (**right**) molecules in a pure POPC (**top**) and POPC:Cholesterol (**bottom**) for both concentration systems with the inhibitor molecules initially in the aqueous phase and lipid phase, as indicated. Regions between phospholipidic polar head groups are shaded in gray. Each different colored line in each plot represents the course of one inhibitory molecule during the simulation time scale of one replica (400 ns simulation production). The other two replicas are presented in the Supplementary Materials, Figures S3 and S4.

We conducted MD simulations for the 12 systems described in Methods. First, from the low concentration systems considered, the binding of each inhibitor to the membrane was followed by measuring the position of the center of mass during the simulation time, as presented in Figure 2. An inhibitor molecule was defined as inserted into the bilayer when its center of mass localized in the region between the average positions of phosphates in each leaflet. Mean insertion fraction of inhibitor molecules in the membrane are shown in Table S2 in the Supplementary Materials. Favipiravir molecules entered the membrane in the timescale of the simulation, localizing mainly in the region of the phosphatidyl choline moiety and not in the predominantly apolar region of the bilayer. It is also noticeable from the trajectories that molecules can then dissolve into the aqueous phase occasionally but return to the bilayer interphase in the scale of the tens of nanoseconds. 

Remdesivir is, on the other hand, a bulky molecule that is not easily incorporated into the bilayer since the insertion–exit events are only infrequently observed. Remarkably, aggregation of the drug molecules was monitored in the bilayer interphase (both from the aqueous or lipidic phase), and this promotes a persistent adsorption. These observations are shared in both high and low concentration conditions. It can be concluded from the time evolution of the center of the mass of the molecules that favipiravir molecules are distributed independently throughout the simulation, whereas remdesivir molecules tend to aggregate.

### 3.2. Drugs Interactions with the Membranes

**Figure 3:**
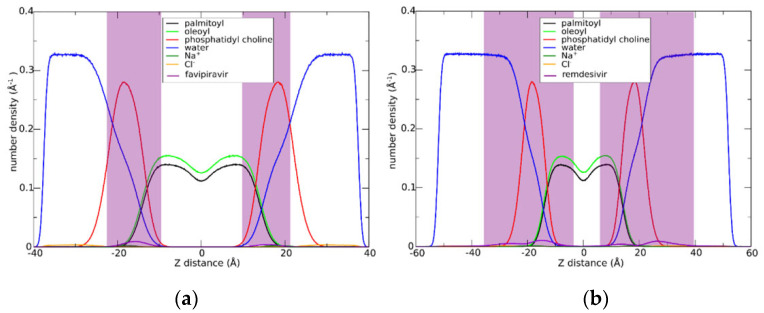
Electron density profiles for all chemical species in the simulated box. Phospholipids were considered within their main components. Shaded regions correspond to distances explored by (**a**) favipiravir and (**b**) remdesivir. Only drug molecules found inserted in the membrane were considered. We used all low concentration trajectories to compute the densities.

Once the compounds were incorporated into the membrane, we proceeded to evaluate the degree of membrane penetration, as shown in the electron density profiles in Figure 3. As can be seen in the figure, the penetration of the compounds is related to their nature and chemical structure. Both results are approximately symmetric for each of the two layers of the simulated membrane system, which also indicates the convergence of the simulation regarding the insertion of the compounds in the membrane. Associated with the considerably smaller size, favipiravir molecules are located at the interface between the phosphatidylcholine heads and the beginning of the fatty acid chains, in a very restricted zone (Figure 3a). Due to the much larger size of remdesivir, the width of the electron density of this compound in the membrane is also more extended, representing the penetration of the molecule in a wider section of the membrane. As seen in Figure 3b, the penetration of remdesivir in each of the layers is extensive, ranging from the outermost zone of the phosphatidylcholine heads, and even outside them towards the aqueous phase, to a deep zone in lipid chains into the membrane. The convergence in the simulations in this case is not perfect, as concluded from the not entirely symmetrical peaks for both layers, an issue that can be explained by the volume, complexity, and flexibility of the inhibitor molecule.

In an attempt to describe the most important intermolecular interactions and the organization of the compounds once inserted into the membrane, we proceeded to calculate the molecular nature of the closest structures to each compound throughout the simulation for both inhibitors. The results are shown in Table 1. As shown, favipiravir molecules are almost exclusively located in a POPC-rich environment, therefore surrounded by POPC molecules (34%) in the case of pure POPC membranes. In cholesterol-containing membranes, cholesterol molecules may be present only in a low proportion (2.4%). Little aggregation of the favipiravir molecules can be detected (0.3% and 0.4%, respectively) in both kinds of membrane assemblies. The corresponding values are decreased in the case of remdesivir, observing a lower percentage of POPC molecules (18.4% and 19.1%, for the pure membranes and POPC:Chol mixture, respectively) in the remdesivir environment. In POPC:Chol membranes, a localization of cholesterol molecules in the close surroundings of a remdesivir molecule is even less likely than in the vicinity of a favipiravir molecule (1.3% vs. 2.4%). On the other hand, the calculated proximity of one remdesivir molecule to another inhibitory unit is substantially higher (4.8% and 7.4% for pure membranes and POPC:Chol, respectively). 

**Figure 4:**
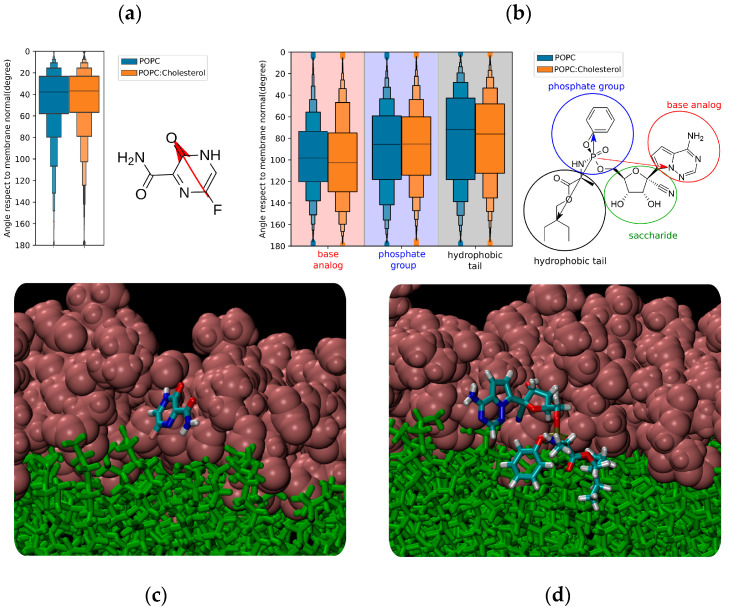
(**a**) Orientation of favipiravir with respect to the bilayer normal in the POPC (**c**) and POPC:Cholesterol (**d**) membranes. Zero degrees means that the O group points towards hydrophilic heads; (**b**) orientation of the different regions of remdesivir, as defined in Figure 1. Three different vectors as described in the figure are used for proper description.

In an effort to define a preferential orientation of the inhibitors in the studied membrane structures, in Figure 4 their localization is explored in the balanced systems during the production time of their simulations. To facilitate interpretation and depict such orientation, we arbitrarily defined vectors for each molecule (one in the case of favipiravir, and three in the case of remdesivir due to its large molecular size and chemical complexity, pointing from the phosphorus atom to the benzyl group, the saccharide-base analog substituent, and the hydrophobic tail). These vectors are shown in Figure 4 and were further evaluated with respect to the normal membrane. As evident from the histograms, favipiravir exhibits a preferential orientation where the F→O vector is located at an angle of approximately 40° to the normal membrane with relatively scarce dispersion. This orientation could be explained due to the favorable interactions and binding of O groups with the hydrophilic phosphate heads of the phospholipids (also shown in Figure 4). On the other hand, remdesivir showed a broader dispersion of the explored orientations. Mean values for the angles describing the position of the base analog, phosphate group and hydrophobic tail were around 100°, 90°, and 70° with respect to the normal membrane, respectively. As depicted in the representative snapshots, such orientation favors the interaction of the remdesivir oxygens with the phosphate heads, while the aromatic components remain inserted within the lipid tails. As noted from the previous analysis, when present, cholesterol molecules approximate the inhibitory compounds. However, as evident from this analysis, the orientation of the drug molecules in both POPC and POPC:Chol systems remains rather similar. 

### 3.3. Effect of Drug Insertion in the Membrane Structure

**Figure 5:**
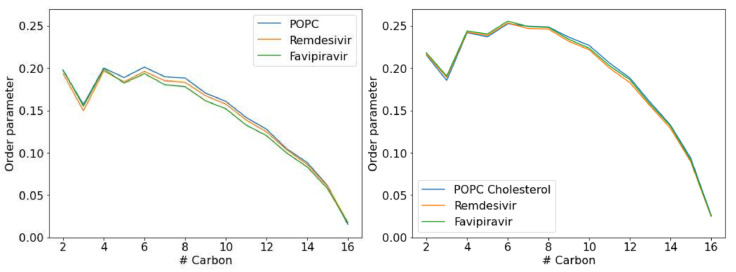
Order parameter of palmitoyl chain hydrogen in the presence and absence of favipiravir and remdesivir in POPC (**left**) and POPC:Cholesterol (**right**) bilayers, calculated under high concentration of drug molecules.

To assess the effect of inserting compounds on the membrane structure, we calculated values for lipid area and order parameters, shown in Table 2 and Figure 5, respectively (area per lipid over simulation production time is shown in Figure S5 in the Supplementary Materials). The presence of favipiravir or remdesivir in the pure POPC membranes does not alter the area per lipid of the same compared to the control, remaining around 64 or 65 ± 1 Å^2^. However, the presence of cholesterol in the membranes yielded a reduction in the area per lipid, from 59.9 to 54.0 and 55 Å^2^ for the presence of favipiravir and remdesivir, respectively. Order parameters represent the effect of the insertion of inhibitor molecules in the packing of the membrane lipid acyl chains. For pure POPC membranes, the presence of either remdesivir or favipiravir does not significantly decrease the order parameter in the middle region of the lipid chains. In a POPC:Chol membrane, the effect of the inhibitor presence is roughly neglectable in terms of acyl chain packing, as derived from the unaltered order parameter profile. One possible explanation for this is the fact that cholesterol molecules, when present, tend to come closer to the inhibitor molecule, moderating its effect on the acyl chain packing. 

## 4. Discussion

The results presented in this study are accompanied by the experimental results presented in a separate paper dealing with the impact of the two drugs, remdesivir and favipiravir, on the properties of different membrane systems [19]. As noted in the introduction, both substances are prodrugs that are further intracellularly metabolized to their active forms yielding the active compounds responsible of their antiviral properties. However, since the prodrugs are the species that primarily interact with the plasma membrane, we have focused here on these chemical species for our study. Both the experimental and the theoretical approach indicate a spontaneous penetration of favipiravir and remdesivir into these lipid bilayers. By comparing the experimental observations and the results of the simulations in further detail, we can draw correlations in terms of: (a) evidence of penetration of the compounds in the membranes; (b) degree of penetration of the compounds in the membranes; (c) differences between the interaction of the compounds in pure POPC membranes and POPC:Chol mixture; and (d) degree of disruption of the structure of the membranes by the presence of the compounds.

We would first like to comment that we are aware that our simulations as well as the associated experiments [19] were performed at comparatively high drug concentrations. The use of such concentrations is required experimentally to obtain sufficiently intense NMR signals. However, during medical use, the plasma concentration of remdesivir immediately after administration might be rather large with about 3 mg/mL and a subsequent rapid decrease within one hour [26]. Therefore, cells might be in contact with large drug concentrations. In order to be comparable to the experimental approach [19], we employed similar conditions in our simulations using a low and a large drug concentration. As results at both concentrations explored in this paper are similar, we assume no great dependence of the investigated parameters on drug concentration apart from the evidenced aggregation.

In agreement with the experimental data, the simulations demonstrate that remdesivir and favipiravir show spontaneous penetration into the model membranes. The transition from the aqueous to the lipid phase occurs under the experimental conditions tested, and lipid embedding is also observed in the simulations in which the compounds start in the lipid phase and remain in this phase at least for the simulation time explored. The partitioning of drug molecules in lipid membranes can be experimentally estimated by octanol-water partition coefficients (logP) [27]. Interestingly, and as we demonstrated in recent work for two other drugs belonging to the family of small-molecule kinase inhibitors, they can have strikingly different logP values spanning several orders of magnitude in the partition equilibrium (ranging from 1.19 to 4.43 for two explored molecules, tofacitinib and lapatinib, respectively) [11,28]. This suggests that although the molecules may exhibit distinctly different membrane interaction patterns based on their respective chemical structures, they all tend to be spontaneously incorporated into the membrane.

Another parameter to consider is the degree of penetration of the explored compounds into the model membranes. As derived from the electron density profiles, the compounds studied were inserted into the membrane in the region close to the phospholipid heads, with a slightly different degree of penetration for each compound. This is in line for remdesivir with cross-relaxation rates measured in ^1^H–^1^H MAS NOESY experiments, which allow the identification of the interaction regions of drugs within the membrane [19]. These results reflect a predominant localization for remdesivir in the glycerol region, almost perpendicular to the normal membrane. In contrast, the preferred orientation of favipiravir molecules in the membrane could not be clearly defined experimentally, although a high mobility throughout the membrane with a slight preference for the glycerol region was found. As presented now in the simulations, the orientation of the compounds in this membrane section allows for interaction with lipid components that retain them in this relatively confined space of each layer.

Even though the presence of cholesterol in the membranes has only a little effect on the final membrane insertion of both studied compounds, some structural changes regarding the presence of it in the membrane could be observed. Moreover, changes in the cholesterol–cholesterol interaction pattern were observed (Figure S6 in the Supplementary Materials). Nevertheless, remdesivir may better accommodate its large structure between the phospholipid heads. As already observed for other compounds, the incorporation of an inhibitor molecule into the upper chain/glycerol/headgroup region should cause a certain space in the inner part of the membrane, which has to be filled by the acyl chain of the surrounding phospholipids, resulting in a slightly lower order parameter for the middle tail region. Once more, such an effect is reduced in the presence of cholesterol, which presents properties in modifying the membrane structure and fluidity, and could eventually moderate the effect of the inhibitor in the membrane packing.

With regard to the physiological relevance, we underline that our study is not aimed to present a mechanism for the antiviral activities of the drugs to explain their efficacy. However, the experimental and theoretical data on lipid membranes may help to understand the cellular uptake mechanism and putative side effects of the molecules. With regard to the latter, a disturbing impact of the drugs on membranes might explain a triggering of apoptosis or decrease of cell viability [29]. Regarding the cellular uptake, to the best of our knowledge it is not known currently, by which process the drugs enter cells. For remdesivir, there are indications for an active import via organic anion transporting polypeptides [30]. However, an incorporation of the drugs into lipid bilayers, as shown in the present study, may hint at a cellular uptake via passive permeation across the lipid phase.

## 5. Conclusions

The present study describes the insertion of two different antiviral drug compounds as a consequence of binding to lipid membranes in accordance with the changes exerted on a model membrane structure and dynamics. It was shown that while remdesivir incorporates into the bilayer causing a disturbance of the membrane structure, favipiravir has no indications for membrane impact. The MD simulation could confirm and well correlate with previous experimental data on an atomistic level.

## Figures and Tables

**Table 1:** Identity of the 10 closest molecules to favipiravir and remdesivir throughout the simulations, presented as percentage. Closest water molecules were omitted.

	Favipiravir		Remdesivir	
	POPC	POPC:Cholesterol	POPC	POPC:Cholesterol
POPC	34 ± 1	27.6 ± 0.4	18.4 ± 0.4	19.1 ± 0.3
Cholesterol	-	2.4 0.1	-	1.3 ± 0.1
Favipiravir/Remdesivir	0.3 ± 0.1	0.4 ± 0.05	4.8 ± 0.2	7.4 ± 0.2

**Table 2:** Membrane area per lipid for pure POPC and POPC:Cholesterol models in the presence and absence of remdesivir and favipiravir.

	POPC (Å^2^)	POPC:Cholesterol (Å^2^)
Favipiravir	64 ± 1	54.0 ± 0.9
Remdesivir	65 ± 1	55 ± 1
control	64 ± 1	59.9 ± 0.7

## Data Availability

When required, raw data of the simulation production files will be available at a provided URL.

## Supplementary Materials

The following supporting information can be downloaded at: https://www.mdpi.com/article/10.3390/membranes12100941/s1, Figure S1: Initial configurations for the simulated assemblies of favipiravir molecules in different model membranes at different concentrations and initial localization of the tested molecules. Figure S2: Initial configurations for the simulated assemblies of remdesivir molecules in different model membranes at different concentrations and initial localization of the tested molecules. Table S1: Final membrane composition of the simulated systems. Figure S3: Position of the center of mass as a function of the simulation time of favipiravir (left) and remdesivir (right) molecules in a pure POPC (top) and POPC:Cholesterol (bottom) for both concentration systems with the inhibitor molecules initially in the aqueous phase and lipid phase, for replica number two. Figure S4: Position of the center of mass as a function of the simulation time of favipiravir (left) and remdesivir (right) molecules in a pure POPC (top) and POPC:Cholesterol (bottom) for both concentration systems with the inhibitor molecules initially in the aqueous phase and lipid phase, for replica number three. Table S2: Mean insertion fraction of tested molecules favipiravir and remdesivir in the membrane considering a mean membrane width for each simulated system. Figure S5: Area per lipid of the POPC (red) and POPC:Cholesterol (blue) membranes containing favipiravir (left) and remdesivir (right). Figure S6: Histogram representing distance between pairs of cholesterol molecules in pure membrane, membrane in the presence of favipiravir (low concentration originally placed inside the membrane) and remdesivir (low concentration originally placed inside the membrane) during the simulation production.
