# Supplementary material for: Effects of the RNA-Polymerase Inhibitors Remdesivir and Favipiravir on the Structure of Lipid Bilayers—An MD Study
Source: Membranes (Basel). 2022 Sep 27;12(10):941. doi: 10.3390/membranes12100941 (PMC9608901; doi:10.3390/membranes12100941)
Supplement: Supplementary file 1 [file membranes-12-00941-s001.zip › membranes-1915857-supplementary.pdf]

membranes

Supplementary Material

Effects of the RNA-polymerase inhibitors Remdesivir and Favipiravir on the structure of lipid bilayers — A MD study

Mauro Bringas[1], Meike Luck[2], Peter Müller[2], Holger A. Scheidt[3,*] and Santiago Di Lella[1,*]

1 nstituto de Química Biológica - Ciencias Exactas y Naturales (IQUIBICEN) - CONICET and Departamento de Química Biológica FCEN, Universidad de Buenos Aires,, Int. Güiraldes 2160, C1428EGA Buenos Aires, Argentina
2 Humboldt Universität zu Berlin, Department of Biology, Invalidenstr. 42, D-10115 Berlin, Germany
3 Leipzig University, Institute for Medical Physics and Biophysics, Härtelstr. 16–18, D-04107 Leipzig, Germany
* Correspondence: santi@qb.fcen.uba.ar (SDL), holger.scheidt@medizin.uni-leipzig.de (HAS)

Citation: Bringas, M.; Luck, M.; Müller, P.; Scheidt, H.A.; Di Lella, S. Effects of the RNA-polymerase inhibitors Remdesivir and Favipiravir on the structure of lipid bilayers - A MD study. *Membranes* **2022**, *12*, 941. https://doi.org/10.3390/membranes12100941

Academic Editor(s): Hugo A. L. Filipe

Received: 30 August 2022
Accepted: 23 September 2022
Published: 27 September 2022

Publisher's Note: MDPI stays neutral with regard to jurisdictional claims in published maps and institutional affiliations.

Copyright: © 2022 by the authors. Licensee MDPI, Basel, Switzerland. This article is an open access article distributed under the terms and conditions of the Creative Commons Attribution (CC BY) license (https://creativecommons.org/licenses/by/4.0/).

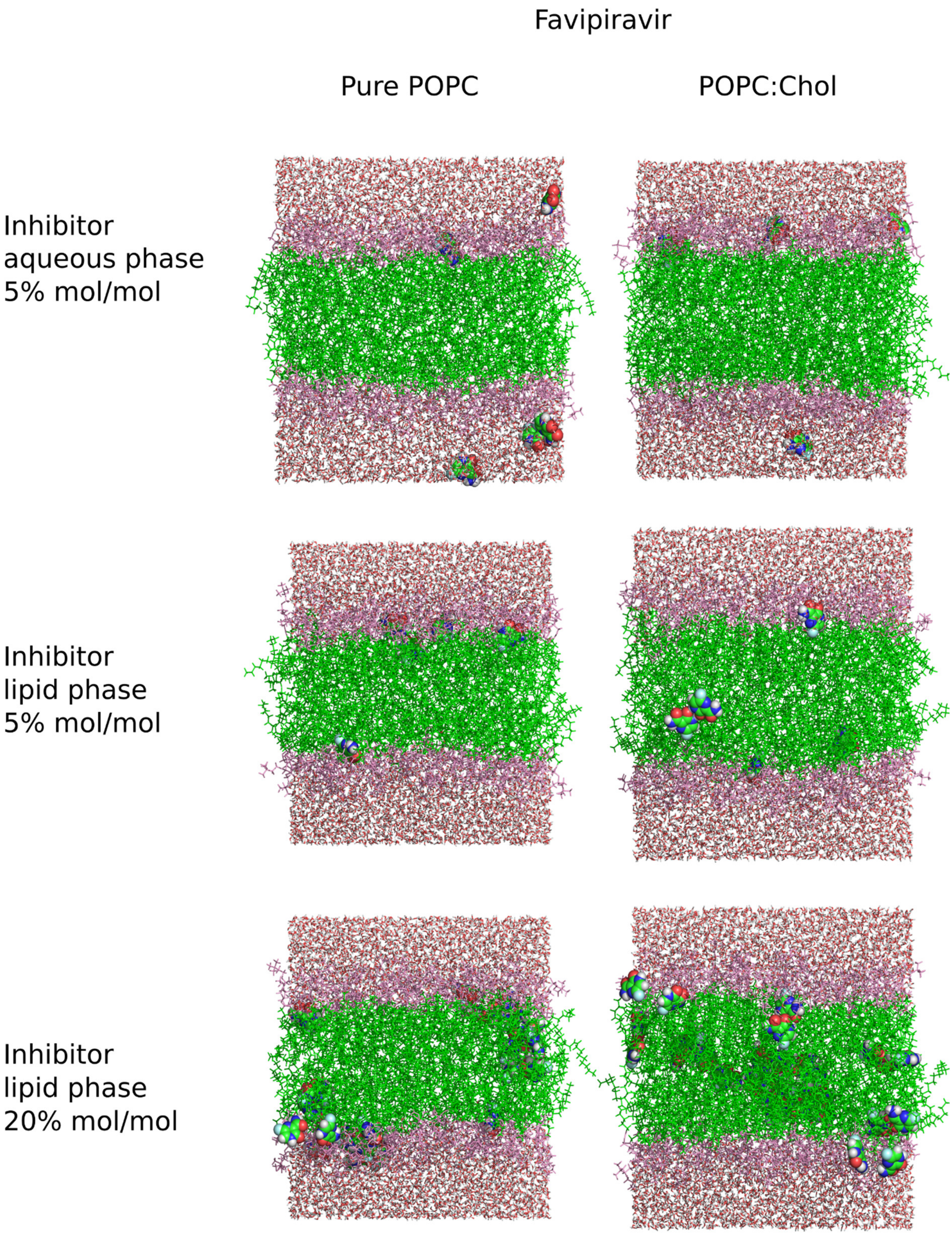

Figure S1. Initial configurations for the simulated assemblies of favipiravir molecules in different model membranes at different concentrations and initial localization of the tested molecules.

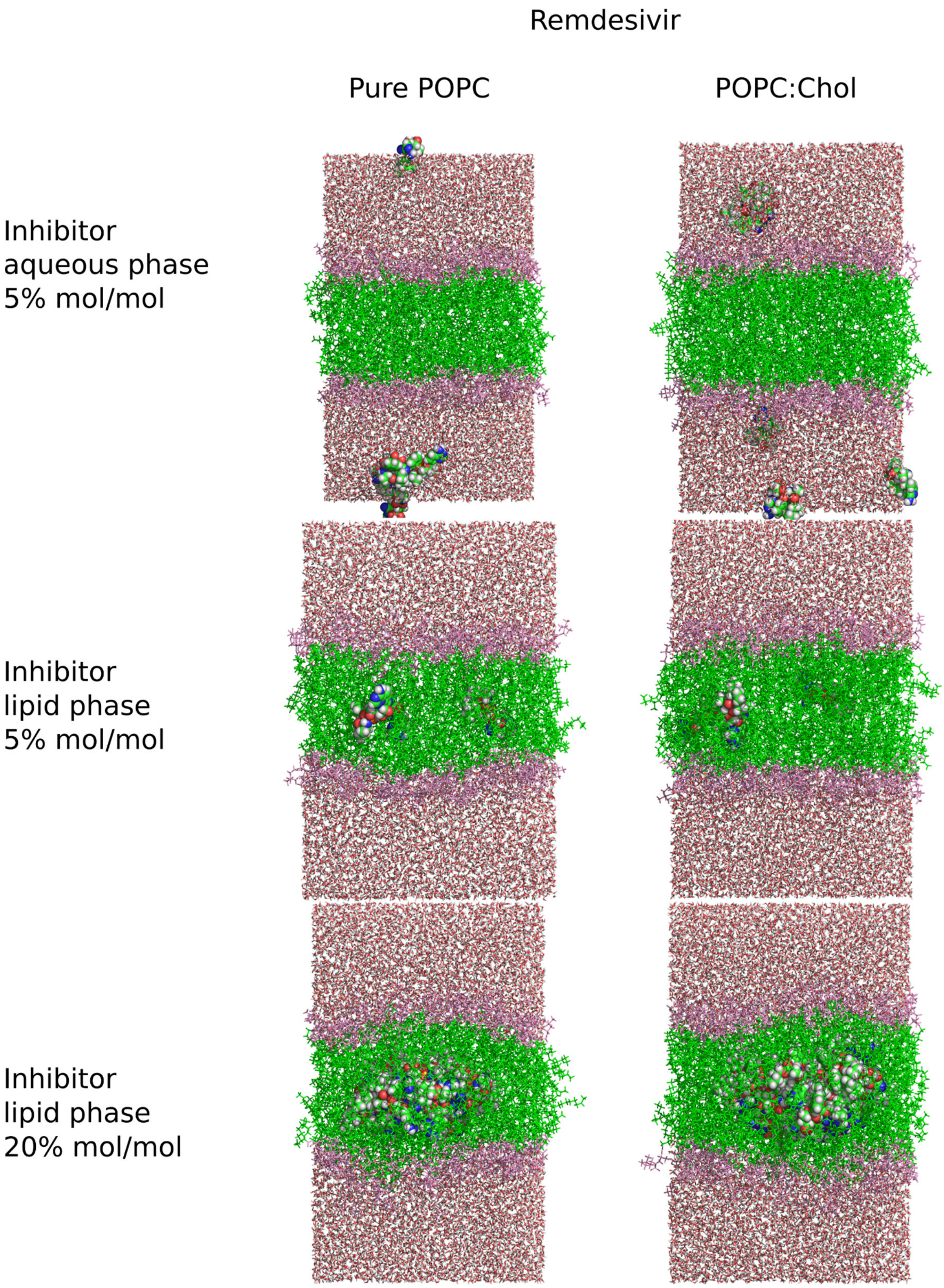

Figure S2. Initial configurations for the simulated assemblies of remdesivir molecules in different model membranes at different concentrations and initial localization of the tested molecules.

Table S1. Final membrane composition of the simulated systems. Number of different chemical species, 1-palmitoyl-2-oleoyl-sn-glycero-3-phosphocholine (POPC), Cholesterol (Chol), Sodium ion (Na^+), Chloride ion (Cl^-), water molecules (WAT), favipiravir molecules (FAV) and remdesivir molecules (REM) are presented for each system.

	**POPC 5% FAV**	**POPC Chol 5% FAV**	**POPC 5% FAV Initial Inside Membrane**	**POPC Chol 5% FAV Initial Inside Membrane**	**POPC 20% FAV**	**POPC Chol 20% FAV**	**POPC 5% REM**	**POPC Chol 5% REM**	**POPC 5% REM. Initial Inside Membrane**	**POPC Chol 5% REM Initial Inside Membrane**	**POPC 20% REM**	**POPC Chol 20% REM**
POPC	128	128	128	128	128	128	128	128	128	128	128	128
Chol		26		26		26		26		26		26
Na^+	12	12	12	12	12	12	12	12	12	12	12	12
Cl^-	12	12	12	12	12	12	12	12	12	12	12	12
WAT	5000	5000	5000	5000	5000	5000	8000	8000	8000	8000	8000	8000
FAV	5	5	5	5	26	26						
REM							5	5	3*	3*	26	26

*Number of molecules was reduced trying to prevent REM aggregation.

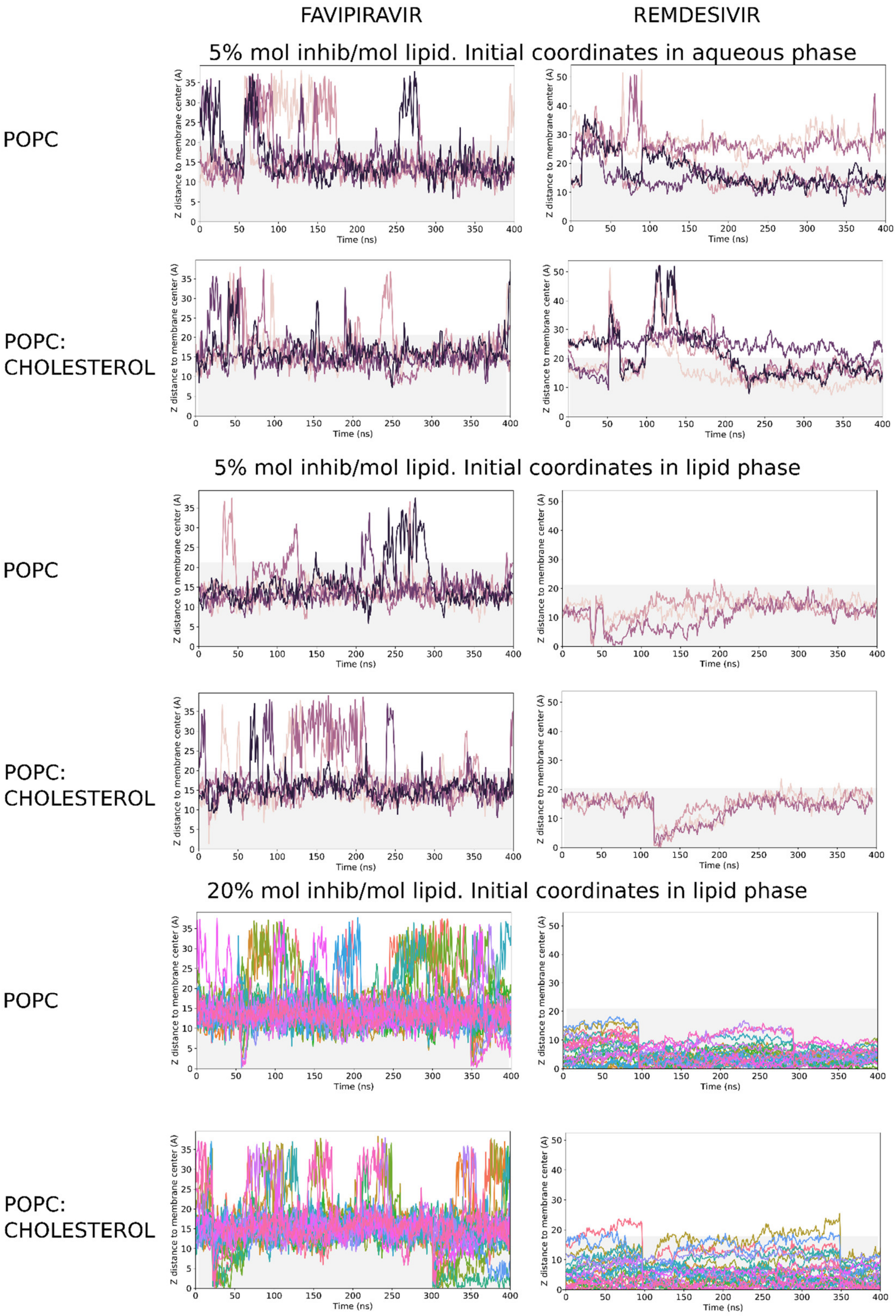

Figure S3. Position of the center of mass as a function of the simulation time of favipiravir (left) and remdesivir (right) molecules in a pure POPC (top) and POPC:Cholesterol (bottom) for both concentration systems with the inhibitor molecules initially in the aqueous phase and lipid phase, as indicated. Regions between phospholipidic polar head groups are shaded in gray. Each different colored line in each plot represents the course of one inhibitory molecule during the simulation time scale, as shown in replica number two.

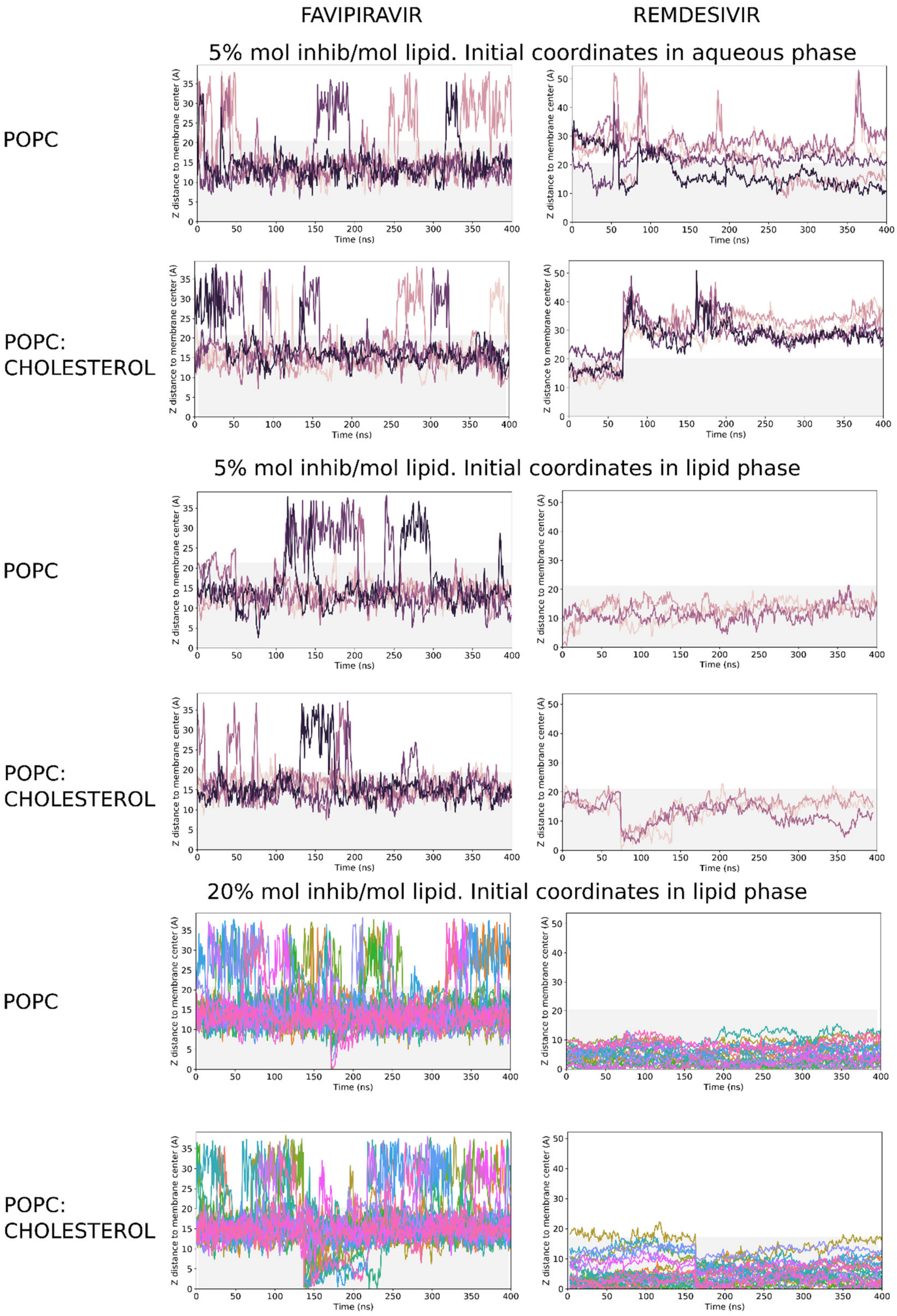

Figure S4. Position of the center of mass as a function of the simulation time of favipiravir (left) and remdesivir (right) molecules in a pure POPC (top) and POPC:Cholesterol (bottom) for both concentration systems with the inhibitor molecules initially in the aqueous phase and lipid phase, as indicated. Regions between phospholipidic polar head groups are shaded in gray. Each different colored line in each plot represents the course of one inhibitory molecule during the simulation time scale, as shown in replica number three.

Table S2. Mean insertion fraction of tested molecules (FAV: favipiravir; REM: remdesivir) in the membrane considering a mean membrane width for each simulated system, as stated.

	**Insertion fraction**
POPC 5% FAV	0.88
POPC Chol 5% FAV	0.88
POPC 5% FAV Initial Inside Membrane	0.91
POPC Chol 5% FAV Initial Inside Membrane	0.90
POPC 20% FAV	0.91
POPC Chol 20% FAV	0.90
POPC 5% REM	0.27
POPC Chol 5% REM	0.30
POPC 5% REM. Initial Inside Membrane	0.60
POPC Chol 5% REM Initial Inside Membrane	0.59
POPC 20% REM	1
POPC Chol 20% REM	0.99

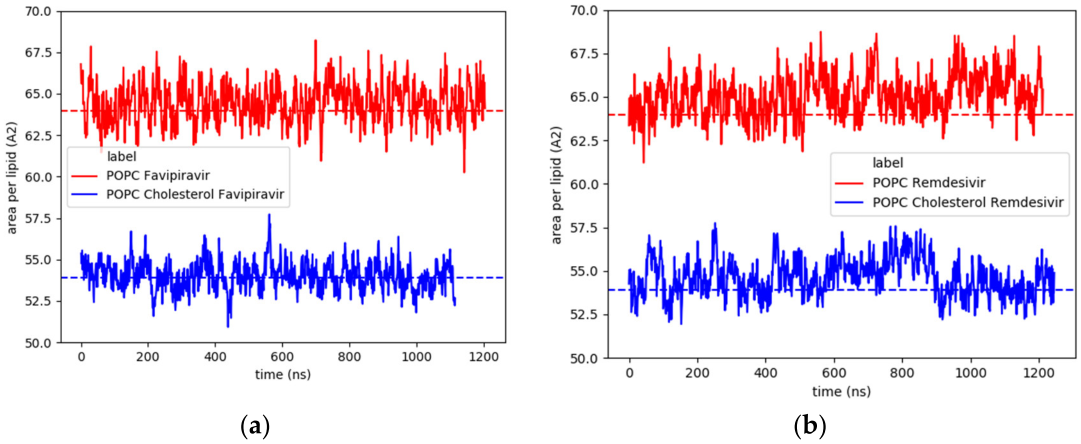

Figure S5. Area per lipid of the POPC (red) and POPC:Cholesterol (blue) membranes containing (**a**) favipiravir (**b**) and remdesivir . In dashed lines, the average value of membrane systems without the drug molecules.

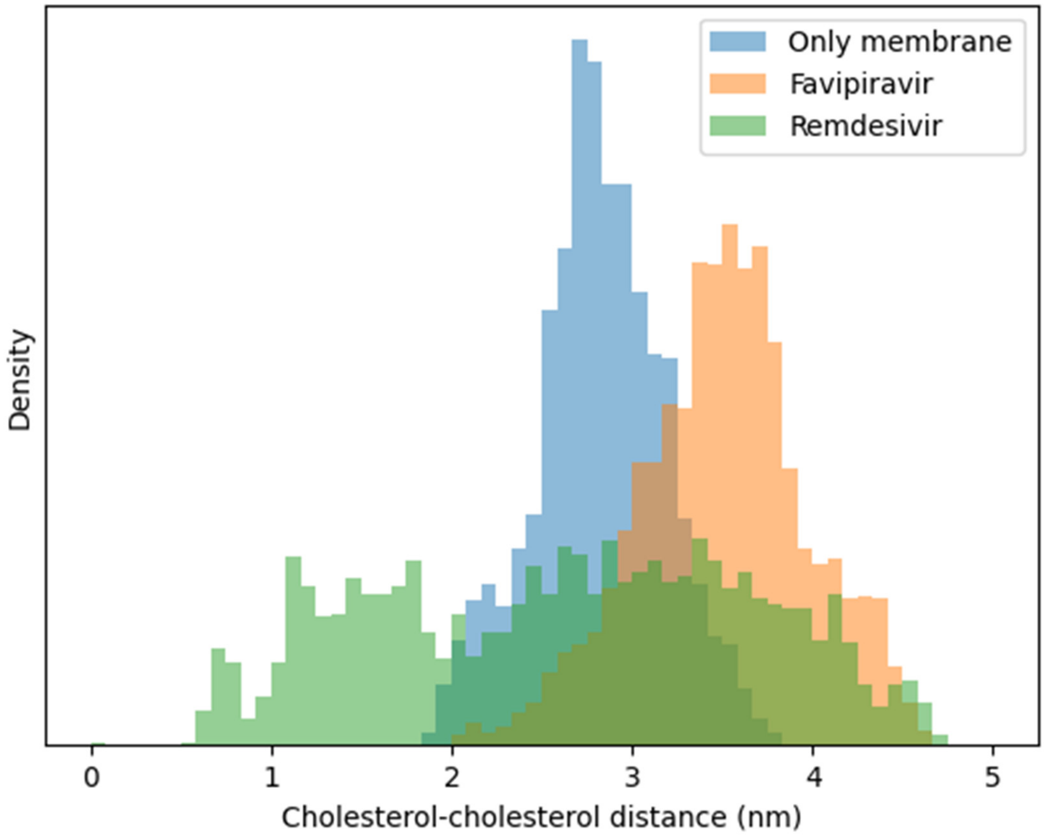

Figure S6. Histogram representing distance between pairs of cholesterol molecules in pure membrane, membrane in the presence of favipiravir (low concentration originally placed inside the membrane) and remdesivir (low concentration originally placed inside the membrane) during the simulation production.
